# Antioxidant and Anti-inflammatory Mechanisms of Neuroprotection by Ursolic Acid: Addressing Brain Injury, Cerebral Ischemia, Cognition Deficit, Anxiety, and Depression

**DOI:** 10.1155/2019/8512048

**Published:** 2019-05-16

**Authors:** Solomon Habtemariam

**Affiliations:** Pharmacognosy Research Laboratories & Herbal Analysis Services, University of Greenwich, Central Avenue, Chatham-Maritime, Kent ME4 4TB, UK

## Abstract

Ursolic acid (UA) is a pentacyclic triterpene which is found in common herbs and medicinal plants that are reputed for a variety of pharmacological effects. Both as an active principle of these plants and as a nutraceutical ingredient, the pharmacology of UA in the CNS and other organs and systems has been extensively reported in recent years. In this communication, the antioxidant and anti-inflammatory axis of UA's pharmacology is appraised for its therapeutic potential in some common CNS disorders. Classic examples include the traumatic brain injury (TBI), cerebral ischemia, cognition deficit, anxiety, and depression. The pharmacological efficacy for UA is demonstrated through the therapeutic principle of one drug → multitargets → one/many disease(s). Both specific enzymes and receptor targets along with diverse pharmacological effects associated with oxidative stress and inflammatory signalling are scrutinised.

## 1. Introduction

**Figure 1:**
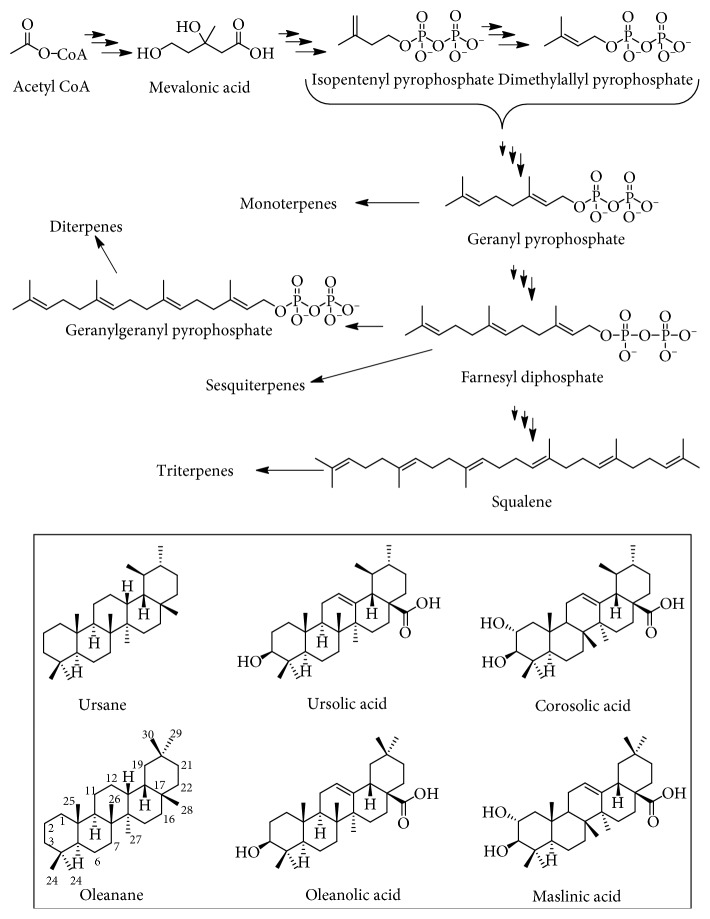
Common biosynthesis pathway of triterpenes including ursolic, oleanolic, corosolic, and maslinic acids.

Ursolic acid (UA) is a common name for the plant triterpenoid compound, 3*β*-hydroxy-12-ursen-28-ic acid. The general biosynthetic routes of terpenoids are depicted in Figure 1 and start from the basic metabolic precursor, acetyl coenzyme A. Since mevalonic acid is an intermediate for terpenoid synthesis, including cholesterols and steroids in animals, this biosynthesis route is also called the mevalonate pathway. The basic building blocks of terpenoids are the five carbon isoprene units that exist in nature in highly reactive isopentenyl pyrophosphate (IPP or isopentenyl diphosphate) and its isomer, dimethylallyl pyrophosphate (DMAPP, dimethylallyl diphosphate). The sequential addition of two, three, and four isoprene units leads to the basic skeletons of the monoterpenoid, sesquiterpenoid, and diterpenoid precursors, respectively, as geranyl, farnesyl, and geranylgeranyl pyrophosphates. All triterpenes are products of two farnesyl pyrophosphate units joined together in head-to-head fashion and trace their acyclic precursor as squalene (Figure 1).

One of the nature's wonder in structural diversity of plant secondary metabolites is reflected through the identification of well over 20,000 triterpenes from just a single squalene precursor. In the first instance, glycosylation to give the diverse saponins and related complex structures is the common route of structural diversity. Beyond oxidations to give the hydroxyl and common carboxylic acid derivatives, cyclisation patterns of terpenoids are the major source of structural diversity. For the triterpenes, the pentacyclic skeletons are among the most common and include the oleanane and ursane groups, among others (e.g., lupanes, gammaceranes, and hopanes). The difference between oleanane and ursane is based on the migration of one methyl group (C-30) in the latter compound from C-20 to C-19 position. The common derivatives of these two compounds are based on C-3 hydroxylation, C-28 carboxylation, and a double bond at C-12 position to form UA and oleanolic acids while further hydroxylation at C-2 position gives corosolic acid and maslinic acid (Figure 1). These two groups of pentacyclic structures both in their aglycone and glyosidic forms are known for a range of biological activities.

Despite its common occurrence in nature including in herbs such as basil, rosemary, and sage or common fruits including apple and pears, UA has diverse pharmacological effects. Some of these effects are reviewed in recent years and include brief overviews of pharmacology related to anticancer [1, 2], antiobesity [3, 4], neurodegenerative, and other diseases [5]. While the anti-inflammatory effect of UA and other triterpenes is widely known [6], it does not possess direct reactive oxygen species (ROS) scavenging effect. This is understandable considering its structure (Figure 1) that lacks the phenolic structural moiety which is often linked to radical scavenging and metal ion chelation pharmacology. A plethora of studies, however, suggest that UA has antioxidant effects in vivo through upregulation of antioxidant defenses. Hence, through the combined antioxidant and anti-inflammatory mechanisms, the compound is endowed with a unique potential to ameliorate a range of neuronal diseases. This review addresses the role of UA in brain injury, cerebral ischemia, cognition deficit, anxiety, and depression through the two common (oxidative and inflammatory) pathological pathways along with specific actions on receptors and enzymes.

## 2. Traumatic Brain Injury and Spinal Cord Injury

Traumatic brain injury (TBI) refers to pathological events related to alteration of the brain function as a result of direct damage by external force such as a blunt to the head. The resulting widespread axonal injuries and lesions in various regions of the CNS could lead to symptomatic abnormalities in cognitive and motor functions, behavioral and emotional domains. The most common and long-lasting abnormality associated with TBI however remains to be memory-related cognitive impairment [7]. Following the primary neuronal tissue damage in TBI, a range of metabolic dysregulation including ischemia, excitotoxicity, calcium, and mitochondrial dysregulations ultimately leads to the inflammation-mediated secondary neuronal damage. Hence, it is reasonable to include anti-inflammatory interventions in the neuroprotective approach of TBI therapy either by pharmacological agents to suppress the overactivated microglia (or astrocytes) [8–10] or by other means such as cell replacement therapies [11] which are yet to be proven to be effective under clinical conditions. The neuroinflammation approach of TBI management should also be seen as targeting the chronic inflammatory events while leaving the normal microglial function intact.

The significance of TBI and, to a lesser extent, spinal cord injury (SCI) has been reviewed in recent years by various authorities. According to the GBD collaborator group study [12], the global incidence of new cases for TBI in 2016 was 27.08 million and SCI was 0.93 million, while their global prevalence was 55.50 million and 27.04 million, respectively. TBI and SCI are classic examples of CNS diseases where new neuronal growth or neurogenesis offers a real potential to regain normal function. Although the significance of neurogenesis in adulthood is well established in experimental animals, there is no consensus for its role in humans. Some evidences show that neurogenesis in the hippocampus of adult humans persists throughout old age [13–15]. On the other hand, the level of neurogenesis has been shown to drastically drop below the detection level even in children [16]. Considering neurogenesis event, particularly at the subgranular zone of the dentate gyrus of the hippocampus, is associated with learning and memory, its role in humans is in need of more research. However, there is still a great deal of enthusiasm in the search of novel compounds that enhance neurogenesis in adulthood as demonstrated for numerous natural products such as curcumin, resveratrol, (-)-epigallocatechin-3-gallate (EGCG), and berberine that increase the production of neurotrophins such as the brain-derived neurotrophic factor (BDNF) or its receptor agonists [17]. Since these natural products are known for their potent antioxidant properties, the dual antioxidant-anti-inflammatory axis along with their multifunctional effects could make them considered as potential therapies for TBI and related pathologies.

The activation and subsequent translocation of the nuclear factor-erythroid 2-related factor 2 (Nrf2) are induced by a variety of stimuli including oxidative stress leading to the transcriptional activation of antioxidant genes such as heme oxygenase-1 (HO-1), GST, NADPH quinone oxidoreductase-1 (NQO-1), and SOD. On the basis that UA exert antioxidative and anti-inflammatory effects on cerebral ischemia by activating the Nrf2 pathway, Ding et al. [18] evaluated the effect of UA on the TBI model in mice (Table 1). In their 24 h assay after TBI induction, administration of UA (50-150 mg/kg) has been shown to reduce brain oedema and neurological insufficiencies. The increased nuclear translocation of Nrf2 protein coupled with the increased expression of NQO1 and HO-1 suggests the role of the antioxidant and/or inflammatory pathway in the neuroprotective effects induced by UA (see also the following section). Moreover, UA was shown to augment the expression level of protein kinase B (Akt), suggesting the activation of the Nrf2 signalling pathway as a mechanism of brain protection under TBI.

Zhang et al. [19, 20] employed a subarachnoid haemorrhage brain injury model in rats to study the potential effect of UA administration (25 or 50 mg/kg) over a 48 h observation period. From the inflammatory end-point measurement that they recorded, a significant reduction of the treatment group included intercellular adhesion molecule-1 (ICAM-1), toll-like receptor 4 (TLR4), nuclear factor-*κ*B (NF-*κ*B) P65, interleukin-1*β* (IL-1*β*), tumor necrosis factor-*α* (TNF-*α*), interleukin-6 (IL-6), inducible nitric oxide synthase (iNOS), and matrix metalloproteinase- (MMP-) 9. These anti-inflammatory effects coupled with a reduction in the apoptosis score suggest the neuroprotective effect of the compound [19]. On the antioxidant line of evidence, the attenuation of early brain injury such as brain oedema, blood-brain barrier disruption, neural cell apoptosis, and neurological deficient by the compound was shown to be coupled with antioxidant levels in the rat cerebral cortex [19]. This include suppression of the malondialdehyde (MDA) levels, increased ratio of reduced (GSH) to oxidized (GSSG) glutathione, recovery of the suppressed superoxide dismutase (SOD), and catalase (CAT) activities, while the markers of apoptosis, caspase-3 and caspase-9 (mRNA and protein levels), were suppressed.

An interesting insight into the neuroregeneration and regrowth potential of UA has been investigated by Sahu et al. [21] using the mouse model of spinal cord injury (Table 1). They have shown that UA (100 or 200 mg/kg) administered 1 h after and then once daily for 6 weeks could promote the recovery of motor functions and axonal regrowth while decreasing astrogliosis. Moreover, UA suppressed the level of proinflammatory cytokines such as IL-6 and TNF-*α* in the injured spinal cord while activating the mitogen-activated protein kinase (MAPK) and phosphoinositide 3-kinase (PI3K)/Akt/mammalian target of rapamycin (mTOR) pathways in the injured spinal cord.

## 3. Cerebral Ischemia

Perhaps the best pathological disease associated with cerebral ischemia is the cerebrovascular disease (stroke) which results from either disruption of blood supply (ischemia) or haemorrhage. Li et al. [22] studied the neuroprotective effect of UA through the transient middle cerebral artery occlusion (MCAO) model of focal cerebral ischemia in mice (Table 1). In the 24 h assay after stroke, administration of 130 mg/kg (i.p.) of UA led to a significant reduction in infarct size coupled with low level of lipid peroxidation (LPO) marker, MDA. In the latter case, the antioxidant effect was coupled with the induction of the nuclear expression of Nrf2 and HO-1 both at the protein and mRNA levels. On the other hand, the cytoplasmic protein level of Nrf2 in ischemic brain at 24 h after MCAO was shown to be suppressed by UA. As an anti-inflammatory agent, treatment with UA also suppressed the level of expression (both at mRNA and protein levels) of TLR4 and NF-*κ*B after stroke in mice. Since Nrf2^−/−^ mice were more susceptible to neuronal damage (infarct size and oxidative and inflammatory scores) in the MCAO model and UA does not extend its protective effect in this mouse group, the Nrf2 signalling pathway is at the forefront of the potential mechanism of action for UA. Hence, the effect of UA is consistent to the well-known effect of the Nrf2-antioxidant response element signalling pathway in oxidative stress [23, 24] and neuroprotection under a variety of CNS disorders including aging [25–28].

In another MCAO model in rats (see Table 1), the beneficial effect of UA in cerebral ischemia and reperfusion-induced brain injury in rats was studied by Wang et al. [29]. When UA was administered (10 or 20 mg/kg, i.g.) at 0.5, 24, and 47 h after reperfusion, decreased neurological deficit scores such as infarct volume and apoptotic cells were observed. The anti-inflammatory mechanism was evident from the reduced level of proinflammatory cytokine concentrations (IL-1*β*, TNF-*α*, and IL-6), TLR4, and inactivated NF-*κ*B, while the high-mobility group box 1 (HMGB1) was also inhibited. In the latter case, HMGB1 is a prominent member of damage-associated molecular patterns (DAMPs) which is released during brain damage, and its binding activity with the DNA is known to induce inflammatory signalling. A number of studies have also shown that HMGB1 can be passively released from necrotic cells or actively secreted in response to inflammatory signals [30]. Working with synergism with the inflammatory pathway, the HMGB1 receptors include the receptors for advanced glycation end products (RAGEs) and toll-like receptor 2 (TLR2) and TLR4. The addition of HMGB-1 in cultured glia and endothelial cells has further been shown to increase the level of TNF-*α* and ICAM-1, respectively, while in the MCAO model, the release of HMGB-1 from neurons at the earliest onset of brain ischemia was demonstrated [31]. Wang et al. [32] also used a MACO model with similar protocol of cerebral ischemia and reperfusion injury and UA administration (5-20 mg/kg, i.g.). They have shown that the improvement in neurological deficit scores such as infarct volume and the number of intact neurons by UA was associated with dose-dependent reduction in the protein levels of MMP2, MMP9, and activated MAPKs, while the level of tissue inhibitor of metalloproteinases 1 (TIMP1) was increased. Furthermore, the protective effect was not only shown to be associated with the increased level of the peroxisome proliferator-activated receptor-*γ* (PPAR-*γ*) protein level and the percentage of PPAR-*γ*-positive cells by UA treatment but also the neuroprotective effect of UA could be ameliorated by a PPAR-*γ* antagonist (bisphenol A diglycidyl ether). Hence, the anti-inflammatory effect of UA as a mechanism includes suppression of the metalloprotease/antimetalloprotease imbalance through action as PPAR-*γ* agonist.

## 4. Cognition Deficit

According to the Alzheimer's Disease International [33], the global figure for dementia in 2015 was 46.8 million people and was estimated to reach close to 50 million people in 2017 with a further projection of doubling every 20 years to reach 75 million by 2030 or 131.5 million in 2050. There is now also over 9.9 million new cases of dementia each year worldwide. The same source put the global cost of dementia as US$818 billion in 2015 or US$ trillion in 2018. The most common form of dementia is Alzheimer's disease (AD) which is an age-related disease characterised by amyloid-*β* (A*β*) plaque deposition at the interneuronal space and neurofibrillary tangles primarily by the microtubule-associated protein, tau, phosphorylation. The therapeutic approaches of dementia are one of the most challenging and what we should still consider at its infancy stage despite the great level of understanding in dementia pathology. Numerous review articles are available in this field (e.g., [34–37]), and herein, the emphasis is to appraise experimental evidences showing pharmacological efficacy for UA. Some of the key findings are summarized in Table 1.

### 4.1. Radio-Induced and Chemically Induced Memory Deficit

The radioprotective effect of UA in mice was evaluated by Tang et al. [38] using the experimental model based on administering 25 mg/kg/daily 1 h after acute (5 Gy) or continuous (0.5 Gy) irradiations for 30 days. They have reported that UA could improve acute irradiation-induced deficits in contextual learning and memory and in novel object recognition memory. The treatment however exacerbated the radiation-induced reduction of neurogenesis in the subgranular zone. Administration of domoic acid in mice can induce cognitive deficits associated with mitochondrial dysfunction which was shown to be ameliorated by UA via modulation of the PI3K/Akt and forkhead box protein O1 (FoxO1) signalling pathways [39]. In this model, FoxO1 activation appear to mediate the mitochondrial dysfunction and memory deficits as FoxO1 knockdown reversed the pathological score induced by domoic acid. Furthermore, the oxidative stress-induced c-Jun N-terminal kinase (JNK) activation and decreased Akt phosphorylation in the hippocampus of domoic acid-treated mice could all be reversed by UA along with the pathological score, promotion of Akt phosphorylation, and FoxO1 nuclear exclusion. As a structural analogue of kainic acid, domoic acid is a natural neurotoxin that induce excitotoxicity in neuronal cells. It is also known to be a toxic agent in the occasional poisoning of people who consume muscles contaminated with the marine planktonic diatom source of domoic acid. The neurological squeal of domoic acid in humans and neurological deficits in experimental animals have already been outlined [40, 41]. In this context, UA has also been shown to protect hippocampal neurons against the kainic acid-induced excitotoxicity [42]. In primary neuronal cultures of cells isolated from the hippocampi of 7-day-old rats, the kainite-induced cell damage and decrease in the mitochondrial membrane potential could be reversed by UA (5–15 *μ*M). In senescent mice subjected to D-galactose-induced neurotoxicity, UA (10 mg/kg, p.o. for 2 weeks) has been shown to improve memory (Morris water maze model) along with increased antioxidant enzymes SOD, CAT, glutathione peroxidase (GPx), and glutathione reductase (GR) and reduction in the level of LPO (MDA) [43]. On the other hand, the activation of caspase-3 in neuronal tissues was suppressed while the level of neural growth-associated protein GAP43 was increased by UA. This data imply that the key regulatory protein for axonal elongation, synaptic plasticity, and nerve sprouting in adult animals that diminishes under experimental dementia could be restored by UA, while apoptosis is suppressed. Related to the age-associated dementia is also the potential effect of UA on the level of antiaging proteins in the brain. Bahrami and Bakhtiari [44] have shown that UA administration (200 mg/kg, i.p for 7 days) could increase sirtuin 1 (SIRT1), SIRT-6, *α*-Klotho, and peroxisome-proliferator-activated-receptor *γ* coactivator 1 beta (PGC-1*β*) protein levels in the isolated hypothalamus (Table 1). While SIRT and PGC-1*β* are known regulators of energy balance such as mitochondrial function, *α*-Klotho is regarded as a hormone that potentially suppress aging as its deficiency is shown to accelerate the degeneration of multiple age-sensitive traits (e.g., [45–48]). The hepatoprotective effect of UA was similarly linked with the generation of the above-mentioned antiaging biomarkers in mice as follows: SIRT1, SIRT6, PGC-1*β*, and Klotho protein expression [49].

The D-galactose-induced neurodegenerative changes were also shown to display the therapeutic potential of UA through antioxidant and anti-inflammatory mechanisms. In addition to improved behavioral performance in mice in the step-through test and Morris water maze task, treatment with UA was shown to decrease advanced glycation end products (AGEs), receptors for AGEs, ROS, and protein carbonyl levels in the prefrontal cortex [50]. As anti-inflammatory compound, UA also suppressed the number of activated microglia cells and astrocytes along with the decreased expression level of adhesion molecule CD11b and glial fibrillary acidic protein, while the expressions of iNOS, cyclooxygenase-2 (COX-2), NF-*κ*B, IL-1*β*, IL-6, and TNF-*α* levels were all shown to be suppressed [50].

The lipopolysaccharide- (LPS-) induced cognitive deficits are one of the best experimental model to show the link between the therapeutic potential of experimental agents in memory function via anti-inflammatory mechanism. In mice treated with LPS, UA (10 or 20 mg, i.p. along with LPS for 8 weeks) was shown to improve cognitive deficits in open field, step-through passive avoidance, and Morris water maze tasks, while the level of proinflammatory markers such as COX-2, iNOS, TNF-*α*, IL-1*β*, IL-2, and IL-6 in the LPS-treated mouse brain was suppressed [51]. Moreover, UA was shown to suppress the LPS-induced I*κ*B*α* phosphorylation and degradation, NF-*κ*B p65 nuclear translocation, and p38 activation in the mouse brain although it did not alter the activation of TLR4, MyD88, ERK, JNK, and Akt.

### 4.2. Diet and/or Diabetes-Induced Memory Deficit

As a model of obesity-induced cognitive impairments, Lu et al. [52] employed a high-fat diet- (HFD-) fed mice to show the promise of UA (10 mg/kg, p.o. for 20 weeks) as a neuroprotective agent. The improvement of the cognition score in both the step-through test and the Morris water maze task was shown to be associated with inhibition of endoplasmic reticulum (ER) stress and I*κ*B kinase *β*/nuclear factor-*κ*B-mediated inflammatory signalling and the restoration of the insulin signalling and PI3K/Akt/mammalian target of rapamycin (mTOR) pathway. Considering the abolition of the observed protective effect by a specific PI3K 110a inhibitor, the classical example of improvement of the insulin signalling pathway relevant to diabetes and/or obesity-mediated insulin resistance and memory deficit was demonstrated for UA. In fact, one of the best-characterised pharmacological effects of UA is related to amelioration of insulin resistance and antidiabetic properties through a variety of mechanisms. In this regard, UA (10 mg/kg) by its own but even better in combination of metformin (150 mg/kg) has been shown, not only to enhance insulin sensitivity but also to improve cognitive impairment [53]. As one expects, this effect is associated with antioxidant and anti-inflammatory mechanisms.

### 4.3. Amyloid-Beta- (A*β*-) Related Pharmacology

Perhaps one of the most researched and promising proof of concept and also the basis for the spectacular failure rate in the history of drug development is related to amyloid-*β* (A*β*) as a target for AD. The recent disappointment in phase III clinical trials has included major player in the pharmaceutical industry including the Merck, Pfizer, J&J, Eli Lilly, and Roche which all have A*β* as a common target. Numerous explanations may be given for these failures including the patients' heterogeneity in disease pathology, the rather long duration of the disease pathology, A*β* may not even be correlating with disease pathology in some cases, or it may even be too late to target it by the time the disease has progressed or the symptom is displayed by patients. The source of dementia, for example, could be of cortical origin which itself could be of AD or frontotemporal dementia; subcortical dementia of Parkinson's disease (PD), Huntington's disease, or Lewy body dementia origin; vascular; or mixed dementia. These different forms might have some common symptoms but differ in their pathological features and hence might not be overcome by common therapeutic approach. As the principal protein component of the senile plaques and overwhelming experimental evidence including animal studies linking A*β* to neurotoxicity and Alzheimer's disease, however, the amyloid hypothesis as a proof of concept is still as attractive as it has ever been. Inhibition of A*β* formation, removal or clearance, or neuroprotection approach is therefore worthwhile to evaluate the therapeutic potential of natural products for AD.

When learning and memory deficits in mice were induced by injection of aggregated A*β*25-35 into lateral ventricles of mice, UA (10, 20, or 40 mg/kg, p.o. for 11 days) could reverse the behavioral hallmark of AD (Morris water maze test) along with inhibition of lipid peroxidation (MDA level) and enhancement of the antioxidant (glutathione (GSH)) level in the hippocampus [54]. The inflammatory markers as shown for the reduced level of IL-1*β*, IL-6, and TNF-*α* levels were also ameliorated in the hippocampus. In vitro, ursolic acid isolated from *Corni fructus* was also shown to ameliorate the *β*-amyloid(25-35)-induced toxicity in PC12 cells via modulation of the NF-*κ*B signalling pathway [55]. Hence, the A*β*-induced expression of iNOS and COX-2 was inhibited through blockade of nuclear translocation of the p65 subunit of NF-*κ*B and phosphorylation of I*κ*B*α*, along with reduced ERK1/2, p-38, and JNK phosphorylation. In PC12 neuronal cells, the increase in free radical production, LPO, and apoptosis induced by A*β* could also be reversed by UA isolated from *Origanum majorana* L. [56, 57].

In Chinese hamster ovary cells stably expressing the human CD36, a drug screening programme identified UA as an inhibitor of A*β* protein interactions with its receptor CD36: the interaction of A*β* to CD36 could be inhibited by ursolic acid up to a maximal inhibition level of 64% at 20 *μ*M [58]. Since CD36 is one of the several receptors for A*β* (e.g., [59, 60]), inhibition of A*β* binding by UA could contribute to the memory-enhancing effect of the compound in vivo.

### 4.4. Acetylcholinesterase Inhibition

Chung et al. [61] identified *Origanum majorana* as a promising acetylcholinesterase (AChE) inhibitor from their screening studies on 139 plant species. A further attempt to identify the active principles led to the identification of UA as an active principle which displayed the in vitro IC_50_ value of 7.5 nM in comparison to 1 nM for tacrine. With the hope of increasing potency, UA-derived hydroxyl-propinyl derivatives have been synthesised and 2-methyl-3-oxo-methyl-ursoloate, for example, as an AChE and butyrylcholinesterase inhibitor in the lower micromolar range has been reported [62]. Since AChE inhibition is by far the best characterised targets for the handful of clinically validated therapeutic agents of AD, further research in this area to identify a more potent UA analogues is well justified.

## 5. Depression and Anxiety

The role of inflammation in anxiety and mood disorders has been well established. A review of clinical and translational data has consistently shown that inflammation could induce the effect on the basal ganglia and cortical reward and motor circuits leading to a reduced level of motivation and motor activity, while the effects of proinflammatory cytokines on monoamines and glutamate could affect the anxiety-related brain regions including amygdala, insula, and anterior cingulate cortex [63]. This relationship is even more prevalent under depressive conditions as inflammatory pathways are activated in depressed patients [64] or in experimental animals [65, 66]. The relationship between inflammatory diseases and depression has also been extensively reviewed and includes cytokine-mediated depression development in patients with connective tissues [67], neuroinflammation in cognitive impairment under psychiatric conditions (e.g., major depressive disorder, bipolar disorder, schizophrenia, and posttraumatic stress disorder) [68], central and peripheral inflammation as the link between depressive disorders and metabolic syndrome [69], and anti-inflammatory intervention as means of managing depressive disorders [70]. Oxidative stress is similarly a major component of anxiety pathology although whether it is the cause or a pathological consequence of the disease is still in need of further research. Insight into the evidences for such correlation from experimental and clinical studies has been reviewed [71], and similarly, the role of antioxidant compounds in depressive-like diseases has been appraised [72]. In this context, the effect of UA as anti-inflammatory and antioxidant agent along with other specific effects in depression and anxiety pharmacology is outlined in the following text (see also Table 2).

The anticonvulsant properties of UA have also been shown in various animal models of epilepsy and seizures. The seizure models in mice employed by Nieoczym et al. [73] included the 6 Hz induced psychomotor seizure threshold test, the maximal electroshock threshold (MEST) test, and the timed intravenous pentylenetetrazol (i.v. PTZ) infusion test. They have shown that UA (50 and 100 mg/kg, i.p.) could increase the seizure thresholds in the 6 Hz and MEST tests without affecting the motor coordination and muscular strength in mice. As an active principle of *Nepeta sibthorpii* Bentham, Taviano et al. [74] have also shown that oral administration of UA (2.3 mg/kg) could induce depressant effect on the CNS by reducing spontaneous motor activity and the number and lethality of pentylenetetrazol- (PTZ-) induced seizures. On the other hand, inhibition of the acetic acid-induced abdominal constriction was also shown for the same dose, while a higher dose (20 mg/kg) increased the reaction time in the hot-plate test (effect reversed by naloxone-opioid receptors mediated) suggesting analgesic effect.

Considering the role of monoaminergic neurotransmitters (serotonin, norepinephrine, and dopamine) in the mood physiology and depressive-like pathologies, attempts have also been placed to characterise the involvement of various neurotransmitter pathways in UA's pharmacology. It is also worth noting that our current pharmacotherapy options for depression are largely based on enhancement of the monoamine transmission system by using selective neuronal reuptake inhibitors of key transmitters such as serotonin or noradrenaline or inhibition of monoamine transmitters degrading enzyme, monoamine oxidase (MAO). The antidepressant therapeutic approach also involve the N-methyl-d-aspartate (NMDA) receptor antagonism with the classical example of ketamine as antidepressant agent. The opioid system has been shown to be involved in the antidepressant-like effect of agents such as folic acid [75] or in the antinociceptive mechanisms of various antidepressants [76].

The tail suspension test (TST) and forced swimming test (FST) are two classical animal models of antidepressant-like effect evaluations where UA showed potent activity. The study by Machado et al. [77], for example, reported a reduction in immobility time in the TST (0.01 and 0.1 mg/kg, p.o.) and in the FST (10 mg/kg, p.o.) in a similar manor to the positive controls, fluoxetine (10 mg/kg, p.o.), imipramine (1 mg/kg, p.o.), and bupropion (10 mg/kg, p.o.). Moreover, by using selective receptor antagonist in the TST, they have shown that UA could induce antidepressant-like effect via the dopaminergic system through dopamine D_1_ and D_2_ receptor activation [77]. In a similar study using TST, Colla et al. [78, 79] demonstrated that the serotonergic and noradrenergic systems (but not the glutamatergic or opioid systems) are also involved in the antidepressant-like effect of UA. This is despite the fact that UA is known to induce antinociceptive effect including through modulation of TRPV1 receptors (antagonism), cGMP generation, and a serotonergic (5HT_1A_ receptor) synergism [80].

The antidepressant-like effects of UA in mice were also demonstrated at just a small dose as 0.1 mg/kg and even through an oral route [81]. Through the TST, the anti-immobility effect of UA was shown to be abolished by a protein kinase A (PKA) (H-89), Ca^2+^/calmodulin-dependent protein kinase II (CAMK-II) (KN-62), protein kinase C (PKC) (chelerythrine), and mitogen-activated protein kinase kinase 1/2 (MEK1/2) (U0126 or PD98059) inhibitors but not PI3K (wortmannin or LY294002) inhibitors. Hence, this data is in agreement with the induction of antidepressant-like effects by drugs through the activation of kinases such as PKA [82], PKC [83], and CAMK-II [84]. Although it does not seem to be involved in the antidepressant-like effect of UA, activation of MEK1/2 is also known to be associated with such mechanism [85].

As one of the active principles of *Artemisia indica*, UA has been shown to display anticonvulsant, antidepressant, and anxiolytic activities in mice. While UA was a positive modulator of *α*1*β*2*γ*2L *γ*-aminobutyric acid (GABA)-A receptor, the observed anxiolytic activity of the compound could be ameliorated by flumazenil suggesting that it acts through the benzodiazepine-binding site of GABA receptors [86]. UA isolated from *P. vulgaris* has also been shown to enhance the pentobarbital-induced sleeping time in mice: an effect which was abolished by GABA receptor antagonist, bicuculline. At fairly small doses (0.03, 0.1, or 0.3 mg/kg) administered prior to pentobarbital, the effect of UA was shown to be associated with activation of GABAA receptors by increasing GABA concentrations in brain tissues [87]. Such effect could have implications for a range of CNS disorders including insomnia. As a key enzyme target for anxiety, epilepsy, and other neurological disorders, the GABA transaminase (GABA-T) was shown to be inhibited by ursolic acid (UA) although the effect observed at the concentration of 100 *μ*g/mL was rather weak [88].

In mice treated with UA (0.1, 1, or 10 mg/kg, p.o.), anxiolytic-like effect has been reported for the highest dose with increased total time in the center, decreased number of rearing responses in the open-field test, and an increased percentage of entries and total time spent in the open arms of elevated plus maze. This effect was also reported to be similar with diazepam (2 mg/kg, p.o.) [79]. Finally, in silico studies have shown a nonselective antidepressant action with strong binding affinity towards MAO-A protein [89] which might have some implication in cellular/animal models. UA also exhibited a significant inhibition of dopamine *β*-hydroxylase (214 *μ*mol/L), weak inhibition of MAO-B (780 *μ*mol/L), and no inhibition against MAO-A [90]. The high concentration used in this study to demonstrate pharmacological efficacy however seems to be therapeutically irrelevant. Overall, UA has both specific mechanisms of receptor and/or enzyme-mediated effects relevant to depression, anxiety, and related psychological disorders that may combine with known antioxidant and anti-inflammatory mechanisms of action in these diseases.

## 6. Parkinson's Disease (PD)

The pathological sequel of neuroinflammation and oxidative stress is best represented by Parkinson's disease (PD) which is characterised by the progressive loss of dopaminergic neurons in the substantia nigra region of the brain along with the formation of intracellular Lewy bodies (LBs) in viable neurons. The production of excessive amount of proinflammatory cytokines such as IL-1*β* and TNF-*α* along with ROS from overactivated microglial cells has long been known to orchestrate the PD pathology (e.g., [91–94]). Other classical inflammatory mediators include iNOS and COX-2 [95–97], p38 MAPK, and NF-*κ*B pathway [97, 98]. The interlinking NF-*κ*B and p38 MAPK signalling pathways are also involved in both the oxidative stress and inflammatory components of neuronal damage in PD [99, 100]. PD is therefore an ideal CNS pathology to demonstrate the therapeutic potential of UA that display anti-inflammatory and antioxidant activities in various animal models.

Animal models of PD are based on induction of oxidative stress and mitochondrial dysfunction by neurotoxins such as MPTP, rotenone, and paraquat. Upon entry into the CNS, MPTP is converted to MPP^+^ via action by monoamine oxidase B (MAOB). The transport and subsequent accumulation of MPP^+^ in dopaminergic neurons leading to amelioration of the mitochondrial electron transport system through interaction with complex I attribute to induction of PD in experimental animals. Hence, MPP^+^ diminishes the mitochondrial power to generate ATP [101] and rather augment the formation of ROS such as superoxide anion. As a therapeutic intervention strategy, antioxidant mechanisms as well as inhibition of the MAOB activation which itself is dependent on the p38 MAPK pathway could be targeted. Some key references of the MPTP-induced PD model and ROS [102, 103] and MAPK [104] signalling pathways are available.

The MPTP-induced PD mouse model has been used to study the effect of UA (5 mg/kg, 25 mg/kg, and 50 mg/kg, p.o. for 21 days). The treatment at the most effective dose of 25 mg/kg was shown to improve behavioral deficits, restored altered dopamine level, and protect dopaminergic neurons in the MPTP-intoxicated mouse [105]. It also ameliorated the MPTP-induced increase of MDA and nitric oxide (NO) levels which is in line with the antioxidant profile of UA in other CNS pathologies.

## 7. General Antioxidant and Anti-inflammatory Effects of UA

Direct ROS scavenging or their formation through metal chelation is a structural attribute endowed by phenolic compounds. Accordingly, compounds that possess the catechol functional group such as caffeic acids [106] and their derivatives including rosmarinic and salvianolic acids [107] or flavonoids such as rutin [108] and others [109] have been reported for their therapeutic potential in AD. Terpenoids could also share this function if they are to become aromatized and carry the phenolic structural moieties as shown for rosemary diterpenoids [110]. On the other hand, terpenoids even as small molecular size as monoterpenes could have antioxidant effects in vivo by inducing antioxidant defenses such as SOD, CAT, GPx, and GR as demonstrated for their therapeutic potential in AD through a variety of assay systems [111]. These terpenoids and even the polyphenolic compounds that display antioxidant effects do also possess anti-inflammatory properties. Hence, they are working to tackle complex diseases through what has been described as one drug → multitargets → one/many disease(s) therapeutic principle [112]. In this context, the therapeutic potential of UA as a prototype lead is shown in various CNS diseases primarily through antioxidant and anti-inflammatory mechanisms and also specific actions in various receptor/enzyme systems outlined in the preceding sections. Similarly, the antioxidant-anti-inflammatory axis has been shown to play a role in the antidiabetic effect of UA as demonstrated in the streptozotocin-induced rats [113, 114] in the db/db diabetic mouse model [115], other models of diabetes nephropathies [116, 117], diabetic-induced monocyte dysfunction and atherosclerosis in mice [118], aortic injury in STZ-induced diabetic rats [119], or clinical trial in human [120]. The anti-inflammatory effect of UA in HFD-induced obese rats [121, 122], inhibition of lipoxygenase-1- (LOX-1-) mediated ROS generation and NF-*κ*B activation as well as atherosclerosis development in mice [123], inhibition of matric metalloproteases in the aortic smooth muscle cells [124], cytokine-induced glioma cell invasion in the transwell cell migration assay [125], or cytokine expression in a macrophage and inhibition of atherosclerosis in mice [126] have also been shown.

The antioxidant and anti-inflammatory effects of UA were demonstrated in hepatoprotection through multiple pathways including antihyperlipidemic effect [127], the carbon tetrachloride- (CCl_4_-) induced liver damage in mice [128–130], high choline diet-induced liver toxicity and endothelial dysfunction [131], ethanol-mediated experimental liver damage in rats [132], liver transplantation model in pigs [133], and LPS-induced hepatocyte damage [134]. The reno- and cardioprotection of UA were similarly evident as demonstrated in the hypoxia-reoxygenation-induced myocardial injury cellular model in H9c2 cells [135], ischemia/reperfusion-induced acute kidney injury in rats [136], and chronic ethanol-induced oxidative stress in the rat heart [137].

**Figure 2:**
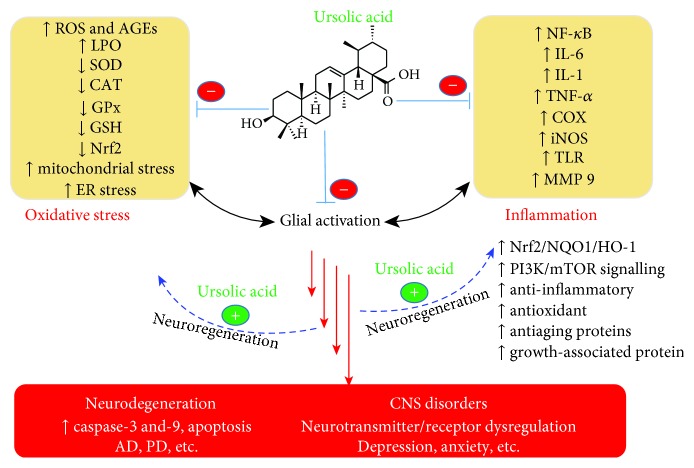
Anti-inflammatory and antioxidant mechanisms of neuroprotection and neuronal function by ursolic acid. Glial cells play a pivotal role in oxidative stress and neuroinflammation that are prevalent in various neurodegenerative disease, traumatic brain/spinal cord injuries, and psychological disorders. By suppressing the generation of ROS, AGEs, and lipid peroxidation (LPO) products as well as increasing antioxidant defenses including through upregulation of the Nrf2 pathway, UA display neuroprotective effects in neuronal cells. The anti-inflammatory action of UA such inhibition of key inflammatory cytokines via the NF-*κ*B signalling pathways is also inhibited by UA. By acting on multiple targets and promotion of neuronal regenerations, UA has diverse function in the CNS. Symbols indicate the following: (-) inhibition and (+) promotion.

UA ameliorates autoimmune arthritis [138], acute inflammation and adjuvant-induced chronic arthritis induced by zymosan in mice [139], or chronic constriction injury-induced neuropathic pain in rats [140]. Other anti-inflammatory and antioxidant actions of UA were in the mouse model of allergic asthma [141], cigarette smoke-induced emphysema in rats as chronic obstructive pulmonary disease (COPD) [142], and the LPS-induced lung injury in mice [143]. The 2,4,6-trinitrobenzenesulfonic acid- (TNBS-) induced colitis (colon shortening and myeloperoxidase (MPO) activity) model in the mouse model [144]; sepsis-induced acute kidney injury via inhibition of ROS and inflammatory cytokines, including TNF-*α*, IL-1*β*, and IL-6 in the kidney from septic mice [145]; sepsis induced in rats by cecal ligation and puncture [146]; LPS-induced acute inflammation model [147]; and various other multiple mechanisms including NF-*κ*B and STAT3 inhibition [148] have been demonstrated. All these data support the anti-inflammatory mechanisms through inhibition of key inflammatory cytokines, COX and iNOS expressions, and antioxidant mechanisms including the activation of the Nrf2 pathway. Hence, the argument for the inflammatory and antioxidant mechanisms of neuroprotection by UA is also supported through the plethora of other systemic effects of UA in various experimental models. An overview of UA's action in CNS disorders is depicted in Figure 2.

## 8. Conclusions

Both as a component of common fruits, herbs, and medicinal plants as well as dietary supplements, UA is a natural product that has been safely used by humans in various forms. Among the plethora of pharmacological effects shown for UA is anti-inflammatory and antioxidant mechanisms in cellular and animal models. In parallel with its effects as antidiabetic, antiobesity, antihyperlipidemic, and hepato-, cardio-, and renoprotective agent and in chronic inflammation (arthritis, long injury, sepsis, and colitis) models, the CNS effect of UA has also been demonstrated. The brain injury, cerebral ischemia, cognition deficit, anxiety, and depression are used in this communication to appraise the therapeutic potential of UA. The antioxidant and anti-inflammatory mechanisms play a pivotal role for UA's effect while other mechanisms include specific effect on neurotransmitter uptake, receptor modulation, and enzyme inhibition, primarily MAO and AChE. In view of such a diverse pharmacological effect/efficacy, a further lead optimisation study by using UA as a prototype drug candidate is well merited.

## Figures and Tables

**Table 1:** Neuroprotective effects of ursolic acid in cellular and animal models.

Model	Procedure	Dosage	Main outcome	Reference
TBI	Wild-type and Nrf2^(−/−)^ mice	50, 100, or 150 mg/kg, i.p.	Neuroprotective in wild-type not Nrf2^(−/−)^ mice; increase the expression of Akt (in Nrf2 upstream signalling)	Ding et al. [18]

Subarachnoid haemorrhage (SH)	Endovascular puncture model in rats	25 or 50 mg/kg, i.p. at 0.5, 24, and 47 h after SH	Decrease the expressions of ICAM-1, TLR4, NF-*κ*B P65, IL-1*β*, TNF-*α*, IL-6, iNOS, and MMP-9; ameliorate apoptosis (TUNEL method); attenuate early brain injury (brain oedema, BBB disruption, neural apoptosis, and neurological deficient)	Zhang et al. [19, 20]

Spinal cord injury (SCI)	C57BL/6J mice	100 or 200 mg/kg, p.o. 1 h after SCI and thereafter once daily for 6 weeks	Promote axonal regrowth and regaining of motor functions; suppress astrogliosis; decrease the levels of IL-6 and TNF-*α*; activate MAPK and PI3K/Akt/MTOR pathways at the SCI	Sahu et al. [21]

Focal cerebral ischemia	Transient MCAO in Nrf2(-/-) and wild-type mice	130 mg/kg, i.p.	Improve neurological deficit and reduce infarct size in wild-type mice; decrease lipid peroxidation; activate Nrf2; decrease TLR4 and NF-*κ*B expression; no effect in Nrf2(-/-) mice	Li et al. [22]

Cerebral ischemia and reperfusion injury	MACO and reperfusion (MCAO/R) in rats	10 or 20 mg/kg, i.g. at 0.5, 24, and 47 h after reperfusion	Decrease neurological deficit scores, infarct volume, and apoptotic cells; suppress IL-1*β*, TNF-*α*, IL-6, TLR4 and HMGB1 levels, and NF-*κ*B signalling	Wang et al. [29]

Cerebral ischemia and reperfusion injury	MCAO/R model in rats	5, 10, or 20 mg/kg, i.g. at 0.5, 24, and 47 h after reperfusion	Reduce the neurological deficit score, infarct volume; increase the number of intact neurons, PPAR*γ* (protein), and PPAR*γ*-positive cells; reduce (protein) MMP2, MMP9, and activated MAPKs; increase TIMP1; effect is dose-dependent	Wang et al. [32]

Radioprotection	Radiation with 5 Gy or fractionated exposure with 0.5 Gy continuously for 10 days in mice; open-field (locomotor) test; novel object recognition test; fear conditioning test; tail suspension test; forced swim test	25 mg/kg/daily, i.p. for 30 days after irradiation	Ameliorate irradiation-induced deficits in contextual learning and memory and in novel object recognition memory; exacerbate radiation-induced reduction of neurogenesis	Tang et al. [38]

Chemical-induced cognitive deficit	Domoic acid-induced cognitive deficit in mice—step-through passive avoidance task; Morris water maze (MWM) test	100 mg/kg, p.o. for 3 weeks	Attenuate the mitochondrial dysfunction and cognitive deficits through promoting Akt phosphorylation and FoxO1 nuclear exclusion in the hippocampus; LY294002, an inhibitor of PI3K/Akt signalling inhibit UA effect	Wu et al. [39]

Chemical-induced neuronal damage	Kainite-induced neuronal damage—primary neuronal cultures of cells isolated from the hippocampi of 7-day-old rats	Pretreatment with 5-15 *μ*M	Suppress neuronal damages; reverse the decrease in mitochondrial membrane potential and free radical generation; effect is dose-dependent	Shih et al. [42]

Aging and chemical-induced neurotoxicity	d-Galactose-induced neurotoxicity in senescent mice; open-field test; Morris water maze	10 mg/kg, p.o. for 2 weeks	Reverse learning and memory impairment; increase the activity of CAT, SOD, GPx, and GR; reduce lipid peroxidation (MDA); inhibit caspase-3 activation	Lu et al. [43]

Aging	Antiaging biomarkers in the hypothalamus of mice	200 mg/kg, i.p. twice daily for 7 days	Increase protein levels of SIRT1 (∼3.5 ± 0.3 folds), SIRT-6 (∼1.5 ± 0.2 folds), *α*-Klotho (∼3.3 ± 0.3), and PGC-1*β* (∼2.6 ± 0.2 folds)	Bahrami and Bakhtiari [44]

Aging	Antiaging biomarkers in hepatic tissues of mice	200 mg/kg, i.p twice daily for 7 days	Increase protein levels of SIRT1 (~5 ± 0.2 folds), SIRT6 (~8 ± 0.5 folds), and PGC-1*β* (~7 ± 0.4 folds)	Gharibi et al. [49]

Inflammatory response in the mouse prefrontal cortex	D-Galactose-induced inflammatory in mice—step-through test and Morris water maze task	10 mg/kg, p.o. for 8 weeks	Decrease AGEs, ROS, and protein carbonyl levels; suppress microglia cells and astrocyte activation; decrease CD11b and glial fibrillary acidic protein expression; suppress iNOS, COX-2, IL-1*β*, IL-6, and TNF-*α* d levels in the prefrontal cortex; attenuate the AGE-induced RAGE expression and NF-*κ*B p65 nuclear translocation	Lu et al. [50]

Cognition impairment	LPS-induced cognitive deficits in mice in open field, step-through passive avoidance, and Morris water maze task	10 or 20 mg/kg, i.p. for 12 weeks	Improve cognitive deficits; decrease the level of COX-2, iNOS, TNF-*α*, IL-1*β*, IL-2, and IL-6; inhibit the induced I*κ*B*α* phosphorylation and degradation, NF-*κ*B p65 nuclear translocation, and p38 activation	Wang et al. [51]

Obesity-induced cognitive impairments	C57/BL6J mice fed a HFD in both the step-through test and the Morris water maze task	10 mg/kg, p.o. for 20 weeks	Improve behavioral performance; inhibit ER stress and I*κ*B kinase *β*/NF-*κ*B signalling; restore insulin signalling and PI3K/Akt/mTOR pathway; effect inhibited by PI-103 (PI3K 110*α* inhibitor)	Lu et al. [52]

Insulin resistance and chronic restraint stress- (CRS-) induced behavioral alterations	Chronic restraint stress (CRS) in mice under insulin resistance—Morris water maze test	5 or 10 mg/kg, p.o. for 30 days	Improve cognitive impairment; decrease serum corticosterone and TNF-*α* levels; improve insulin sensitivity, learning, and cognitive performance; synergize with metformin	Mourya et al. [53]

*β*-Amyloid-induced memory impairment	Intracerebroventricularly administered A*β*(25-35) in mice—open-field test and Morris water maze test	10-40 mg/kg, p.o. for 11 days	Reverse learning and memory deficits; suppress MDA, IL-1*β*, IL-6, and TNF-*α* and increase GSH levels in the hippocampus	Liang et al. [54]

*β*-Amyloid-induced neurotoxicity	PC12 cells subjected to A*β*(25-35)-induced toxicity	Up to 250 *μ*M	Inhibit the expression of iNOS and COX-2; block NF-*κ*B nuclear translocation (p65 subunit); reduce I*κ*B*α*, ERK1/2, p-38, and JNK phosphorylations; inhibit ROS generation and cell death	Yoon et al. [55]; Heo et al. [56]; Hong et al. [57]

*β*-Amyloid interactions with its receptor CD36		Up to 20 *μ*M	Block the binding of A*β* to CHO-CD36 cells or A*β* to microglial cells; reduce subsequent ROS production	Wilkinson et al. [58]

Abbreviations: AGEs: advanced glycation end products; Akt; protein kinase B; BBB: blood-brain barrier; CAT: catalase; COX: cyclooxygenase; ER: endoplasmic reticulum; ERK: extracellular signal-regulated kinase; GPx: glutathione peroxidase; GR: glutathione reductase; GSH: glutathione; HMGB1: high-mobility group protein B1; ICAM-1: intercellular adhesion molecule-1; I*κ*B*α*: nuclear factor of kappa light polypeptide gene enhancer in B-cell inhibitor, alpha; IL-1*β*: interleukin-1*β*; IL-6, interleukin-6; iNOS: inducible nitric oxide synthase; i.g.: intragastric; i.p.: intraperitoneal; JNK: c-Jun N-terminal kinases; LPS: lipopolysaccharide; MACO: middle cerebral artery occlusion; MAPK: mitogen-activated protein kinase; MMP: matrix metalloproteinase; MDA: malondialdehyde; mTOR: mammalian target of rapamycin; Nrf2: nuclear factor-erythroid 2-related factor 2; NF-*κ*B: nuclear factor-*κ*B; PGC-1*β*: peroxisome proliferator-activated receptor-gamma coactivator-1*β*; PI3K: phosphoinositide 3-kinase; p.o.: per os or oral administration; PPAR*γ*: peroxisome proliferator-activated receptor-*γ*; RAGE: receptor for advanced glycation end products; ROS: reactive oxygen species; SCI: spinal cord injury; SH: subarachnoid haemorrhage; SIRT: sirtuin; SOD: superoxide dismutase; TBI: traumatic brain injury; TLR4: toll-like receptor; TIMP1: tissue inhibitor of metalloproteinase 1; TNF-*α*: tumor necrosis factor-*α*.

**Table 2:** Effect of ursolic acid in behavioral animal models.

Model	Procedure	Dosage	Main outcome	Reference
Sedative, anticonvulsant, and analgesic effect	Lethality of pentylenetetrazol-induced seizures; writhing test and the hot-plate test of nociception in mice	2.3 or 20 mg/kg, p.o.	Inhibit the acetic acid-induced abdominal constriction; increase (higher dose) the reaction time in the hot-plate test; effect involves opioid receptors (reversed by naloxone)	Taviano et al. [74]

Antidepressant-like effect	Tail suspension test (TST) and the forced swimming test (FST) in mice	TST (0.01 and 0.1 mg/kg, p.o.) and in the FST (10 mg/kg, p.o.)	Reduce the immobility time in the TST and FST; effect abolished by a dopamine D(1) receptor antagonist (SCH23390)	Machado et al. [77]

Antidepressant-like effect	TST and FST; and open-field test (OFT) (locomotor activity) in mice	0.1 mg/kg, p.o.	No effect on locomotor activity; serotonergic and noradrenergic systems involved in the antidepressant-like effect (receptor antagonism study)	Colla et al. [78]

Anxiolytic-like effects	Open-field test, elevated plus maze test, light/dark box test, and marble burying test in mice	0.1, 1, and 10 mg/kg, p.o.	Anxiolytic-like effect (higher dose)—increased total time in the center, decreased number of rearing responses in the OFT, and an increased percentage of entries and total time spent in the open arms of elevated plus maze; no effect in the light/dark box and marble burying tests	Colla et al. [79]

Antinociceptive activity	Formalin or acetic acid-induced nociceptive response in mice	2 mg/kg, i.p.	Antinociceptive effect; effect mediated through cGMP; additive/synergism with 5HT1A receptors and antagonistic activity towards TRPV1 receptors	Verano et al. [80]

Antidepressant-like effects	TST in mice	0.1 mg/kg p.o.	Anti-immobility effect; effect abolished by the treatment of mice with H-89, KN-62, chelerythrine, U0126, or PD98059, but not with wortmannin or LY294002: activation of PKA, PKC, CAMK-II, and MEK1/2 mediate the antidepressant-like effects	Ramos-Hryb et al. [81]

Insomnia treatment	Pentobarbital-induced sleeping behaviors in mice	0.3 mg/kg, p.o.	Enhance sleep duration in pentobarbital-treated mice; effect attenuated by GABAA receptor antagonist (bicuculline)	Jeon et al. [87]
